# Phase-change materials based on amorphous equichalcogenides

**DOI:** 10.1038/s41598-023-30160-7

**Published:** 2023-02-18

**Authors:** Roman Golovchak, Jarres Plummer, Andriy Kovalskiy, Yuriy Holovchak, Tetyana Ignatova, Anthony Trofe, Bohdan Mahlovanyi, Jozef Cebulski, Piotr Krzeminski, Yaroslav Shpotyuk, Catherine Boussard-Pledel, Bruno Bureau

**Affiliations:** 1 Department of Physics, Engineering and Astronomy, Austin Peay State University, Clarksville, TN 37044 USA; 2 Department of Nanoscience, University of North Carolina, Greensboro, NC 27401 USA; 3 Institute of Physics, University of Rzeszow, 35-959 Rzeszów, Poland; 4 CNRS, ISCR [(Institut des Sciences Chimiques de Rennes)] – UMR 6226, University of Rennes 1, 35042 Rennes, France; 5 Department of Sensor and Semiconductor Electronics, Ivan Franko National, University of Lviv, Lviv, 790017 Ukraine

**Keywords:** Materials science, Information storage

## Abstract

Phase-change materials, demonstrating a rapid switching between two distinct states with a sharp contrast in electrical, optical or magnetic properties, are vital for modern photonic and electronic devices. To date, this effect is observed in chalcogenide compounds based on Se, Te or both, and most recently in stoichiometric Sb_2_S_3_ composition. Yet, to achieve best integrability into modern photonics and electronics, the mixed S/Se/Te phase change medium is needed, which would allow a wide tuning range for such important physical properties as vitreous phase stability, radiation and photo-sensitivity, optical gap, electrical and thermal conductivity, non-linear optical effects, as well as the possibility of structural modification at nanoscale. In this work, a thermally-induced high-to-low resistivity switching below 200 °C is demonstrated in Sb-rich equichalcogenides (containing S, Se and Te in equal proportions). The nanoscale mechanism is associated with interchange between tetrahedral and octahedral coordination of Ge and Sb atoms, substitution of Te in the nearest Ge environment by S or Se, and Sb–Ge/Sb bonds formation upon further annealing. The material can be integrated into chalcogenide-based multifunctional platforms, neuromorphic computational systems, photonic devices and sensors.

## Introduction

Chalcogenide phase-change materials (PCMs) are known for their unique behavior during switching between the amorphous and crystalline states^1–3^. Accompanying pronounced changes in the optical and electronic transport properties happening on a nanosecond timescale had led foundation for many applications of PCMs in data storage devices, reconfigurable meta-optics, optical switches, tunable emitters and absorbers, nonvolatile photonics, even in neuromorphic photonic computing^1–12^. Rapid and reversible transitions between a highly resistive and conductive states (usually resistivity changes by several orders in magnitude) occurring at moderately elevated temperatures is especially intriguing for new-generation memory devices^1,2,13^. To date, research was focused mostly on PCMs from ternary Ge–Sb–Te (GST) compositions with different concentrations of constituent elements, including boundary Ge–Te (such as GeTe) and Sb–Te (eutectic Sb_69_Te_31_, Sb_40_Te_60_ or similar) compounds^1–13^. Recently, introduction of Se into this matrix has led to the discovery of Ge–Sb–Se–Te (GSST) family of PCMs, where satisfactory phase change memory effect in both electrical and optical properties was combined with the advantage of significantly improved glass forming ability and optical transparency in 1.0–18.5 μm wavelengths range^4^. In principle, this result follows the expected behavior when Te is replaced with Se in multinary chalcogenide systems^14–17^. Moreover, one can notice through the analysis of applications and physical properties of various chalcogenides that each chalcogen type (S, Se or Te) brings its own distinguished functionality into the compound^17–20^. Thus, the major driving factors for adding Se into composition are the improved glass-forming ability and generally higher optical transparency^17–19^; Te is known to promote a valence alteration and, therefore, increases the variety of possible structural motives and crystallization affinity^17–21^; S is usually used to improve sensitivity to the external factors or enhance nonlinear optical effects^22–24^. Following this trend, we can argue that including Sulfur into PCM composition along with Se and Te could add new functionalities not explored so far in this class of materials. Such supposition is based on a recent renaissance of antimony and germanium containing sulfides and selenides, which are proposed as perspective medium for switchable, high-saturation, high-efficiency and high-resolution dynamic meta-pixels for enhanced meta-displays (Sb_2_S_3_ and Sb_2_Se_3_)^25^, monocrystalline path formation under laser irradiation (SbSI)^26,27^, 3D waveguides (Ge_23_Sb_7_S_70_)^28^, solid-state lithium batteries (Ga_2_S_3_ modified Ge_33_S_67_)^29^ and glass-on-graphene photonics^30^. Recently, Sb_2_S_3_ and Sb_2_Se_3_ compounds were proven to possess a phase-change memory effect too^31,32^. All these advancements become possible due to the unique physical properties of sulfides, such as relatively wide optical gap, high refractive index, low optical losses and high sensitivity to the external factors. Sulfide-based chalcogenide glasses also possess a satisfactory solubility of various rare-earth ions, which makes them suitable for optical fiber amplifier and energy conversion device applications^17,21,33–35^.

Including all three chalcogens into PCM composition opens a wide range of possibilities for improving and tailoring the medium properties, but simultaneously complicates the understanding of glass structure and, therefore, our ability to develop analytical structural models for the observed effects. As a rule, new multinary compositions are designed keeping the total content of chalcogen atoms (S, Se, Te) at the level of 50 at% or higher, as it is reasonably believed that chalcogens help glass formation due to a steric flexibility of their covalent bonds^18,19^. However, the most interesting phenomena usually occur at the boundaries of glass forming regions, where reversible/irreversible phase changes become possible at relatively low activation energies. Recent discovery of Ag_4_In_3_Sb_67_Te_26_ (AIST) PCM^36,37^, Ovonic switching effect in Ge_60_Se_40_ thin films^38^ or laser waveguide writing in SbSI compound^26,27^ just confirms this prospective.

In this work, we introduce a new class of PCMs based on Sb-rich germanium equichalcogenides (containing equal amount of S, Se and Te) with total content of chalcogen atoms less than 50 at%. The physical properties of the discovered Ge_15_Sb_40_S_15_Se_15_Te_15_ bulk glasses and thin films are investigated and compared to the earlier research on Ge_20_Sb_20_S_20_Se_20_Te_20_ composition from the same Ge–Sb–S–Se–Te equichalcogenide family, in which thin films show a superior photosensitivity to the visible and NIR light in a broad temperature interval without any phase change effects below 200 °C^39^. Just a simple change in the Sb content makes it possible to obtain a phase-change material within the same Ge–Sb–S–Se–Te equichalcogenide family, which testifies a true multifunctionality of the proposed glass matrix. The combination of two materials can be easily done through co-evaporation thin film technique, creating the gradient in Sb concentration through diffusion mechanism or synthesis route, which would open a way to build integrated optical/electronic circuits based on this single-family material. The Sb-rich composition is chosen owing to a growth-dominated crystallization mechanism in Sb-rich PCMs and their high crystallization rates^40^, while Ge is usually added to improve the amorphous phase stability of GST. So far, the phase-change memory effect was never reported for any of the mixed S–Se–Te chalcogenides.

## Material and methods

**Figure 1:**
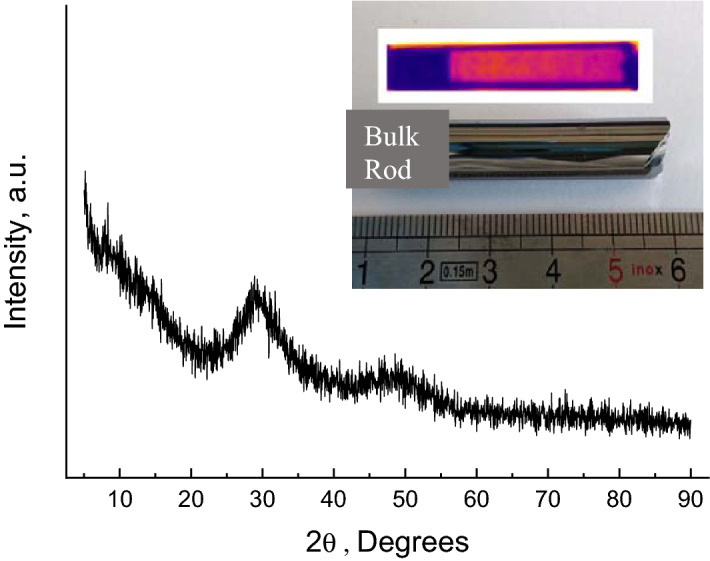
As-prepared Ge_15_Sb_40_S_15_Se_15_Te_15_ bulk glass. Main figure shows featureless XRD pattern verifying vitreous nature of the obtained bulk material. The insert shows an ingot as removed from the ampoule and its IR image, testifying good homogeneity of the prepared bulk glass.

Bulk equichalcogenide Ge_15_Sb_40_S_15_Se_15_Te_15_ glasses were prepared by conventional melt quenching method using 5N-purity elements (Alfa Aesar, Umicore). The appropriate amounts of chemical precursors were vacuum sealed in 10 mm diameter silica ampoules, heated up to 800 °C, homogenized at this temperature for 12 h in rocking furnace and quenched from 600 °C into room temperature water. To relieve the mechanical strains appeared as a result of rapid quenching, the ampoules were additionally annealed at close to glass transition temperature (*T*_*g*_) for 4 h. The as-prepared glass was vitreous in nature, showing no significant reflexes in X-ray diffraction (XRD) patterns (Fig. 1) and uniform infrared (IR) image. XRD spectra were measured with the Rigaku Miniflex 6G system, equipped with an accessory for thin film XRD measurements at different temperatures.

Optical transmission spectra were measured by Agilent Cary 5000 (UV/VIS/NIR) and Bruker vertex 70v (IR) spectrophotometers using 2 mm thick disks polished to a high optical quality.

Thin films were prepared in high vacuum using MBRAUN thermal evaporator and small chunks of bulk Ge_15_Sb_40_S_15_Se_15_Te_15_ glass loaded into tungsten boats as evaporation source. Thickness of the films was monitored using quartz crystal microbalance method implemented into MBRAUN evaporator system. Microscopy glass slides, Si/SiO_2_ chips with interdigitated gold electrodes (*p*-type monocrystalline silicon with 300 nm thick SiO_2_ layer; 30 μm wide, 1500 μm long and 10 nm/100 nm thick Cr/Au finger electrodes; 30 μm line spacing; 20 interdigitated pairs of 40 fingers) and high-density Al_2_O_3_ ceramic chips with interdigitated electrodes (11 pairs of 100 μm wide and 2200 μm long Ti/Cu/Ni/Au fingers with 0.1 μm/10 μm/4 μm/1 μm metal thicknesses respectively; 100 μm line spacing; 22 fingers in total) were used as substrates that were simultaneously mounted on a rotational holder of the evaporator.

Composition of the prepared bulk glasses and thin films was confirmed using a scanning electron microscope (SEM) FEI Helios NanoLab 650 and TESCAN VEGA 3 equipped with an energy-dispersive spectroscopy (EDS) analyzer Bruker XFlash 6/30.

A temperature-controlled chamber, Linkam (L-THMS350/EV-4), was used to mount the films during conductivity and Raman measurements. The chamber was purged with pure nitrogen gas to reduce oxydation processes. Raman spectra were collected at different temperatures using 785 nm laser excitation and LabRam HR800 (Horiba Jobin–Yvon) spectrometer. To avoid possible photoinduced changes, the power of the laser was reduced with optical density filters and did not exceed 1 mW. Conductivity measurements were performed using HIOKI LCR meter in DC mode with a bias voltage of 1 V.

DSC measurements were conducted using a NETZSCH DSC-204 F1 instrument calibrated with a set of standard elements. Bulk ~ 15 mg chunks of glass were heated in 40 μl aluminum crucibles under nitrogen atmosphere at *q* = 2, 5, 10, 15, and 20 K/min constant heating rates. DSC data for thin films were collected on a ~ few mg powders obtained by scraping the as-deposited film from substrate with a hard tool. The DSC scan protocol included two runs at every *q*: the first run revealed a glass-to-supercooled liquid transition and crystallization peaks, while the second one of a fully crystallized sample provided a confident baseline and a check of completeness of crystallization processes. To assure the repeatability of the results, the DSC measurements were repeated at least three times for each *q*, using a fresh sample for every measurement.

XPS spectra were recorded using a high-resolution ESCALAB Xi+ spectrometer (Thermo Electron North America LLC) equipped with a monochromatic Al *K*_*α*_ (1486.6 eV) X-ray source under a vacuum of 10^−8^ Torr (or better). The surface of the samples was cleaned with a quick Ar-ion sputtering to remove surface contaminations directly before the measurements. The surface charging from photoelectron emission was neutralized using a low energy (< 10 eV) electron flood gun. The experimental positions of the core levels were adjusted by referencing to the position of 1 s core level peak (284.6 eV) of adventitious carbon^41^. XPS data were analyzed with standard CASA-XPS software package, using Shirley background and a pseudo-Voigt line shape for the core level peaks^42^. The pseudo-Voigt function was approximated by Gaussian/Lorentzian product form, where the mixing was fixed to be 0.3 (0 is a pure Gaussian, 1 is a pure Lorentzian) for all doublets of the analyzed core-levels. The 3*d* core-level XPS spectra of Se, Te and Ge, 4*d* core-level XPS spectra of Sb and 2*p* core-level XPS spectra of S were used for quantitative analysis of chemical order in the investigated thin films. The number of doublets (which consisted of *d*_5/2_ and *d*_3/2_, or *p*_3/2_ and *p*_1/2_ components owing to spin–orbit splitting) within a given peak was determined by an iterative curve fitting procedure in which a doublet was added only if it significantly improved the goodness of the fit. The parameters used to link the *d*_5/2_ and *d*_3/2_ components were: a peak separation of 0.56 eV for Ge, 1.24 eV for Sb, 0.82 eV for Se, 1.46 eV for Te, and an area ratio 1.45 for all doublets of *d* core levels. For the *p* core level of S, the peak separation was taken to be 1.16 eV and a *p*_3/2_/*p*_1/2_ peak area ratio of 2 was used. The full width at half maximum (*fwhm*) was assumed to be the same for the peaks within a given doublet, but different *fwhm* values were allowed for independent doublets of the same core-level peak. With these constraints, the uncertainties in the peak position (binding energy, BE) and area (*A*) of each component were ± 0.05 eV and ± 2% respectively.

## Results and discussion

**Figure 2:**
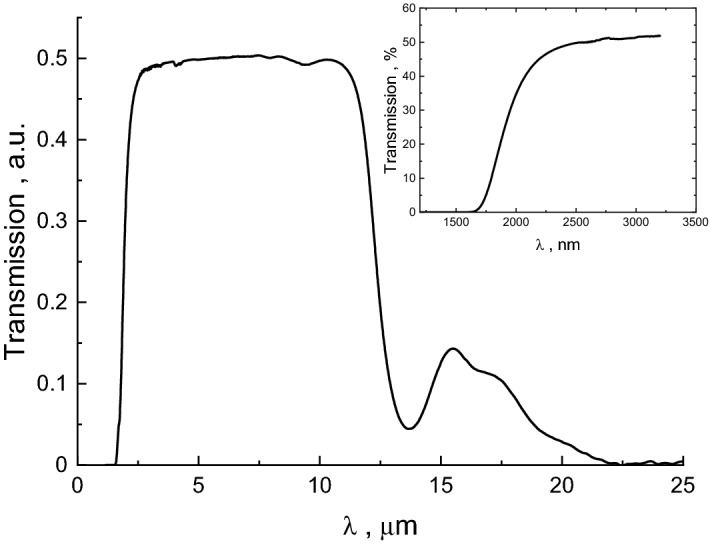
FTIR spectra of as-prepared Ge_15_Sb_40_S_15_Se_15_Te_15_ glass. The glass is transparent within 2–12 μm wavelengths range. The insert shows transmission in the fundamental optical absorption edge region.

**Figure 3:**
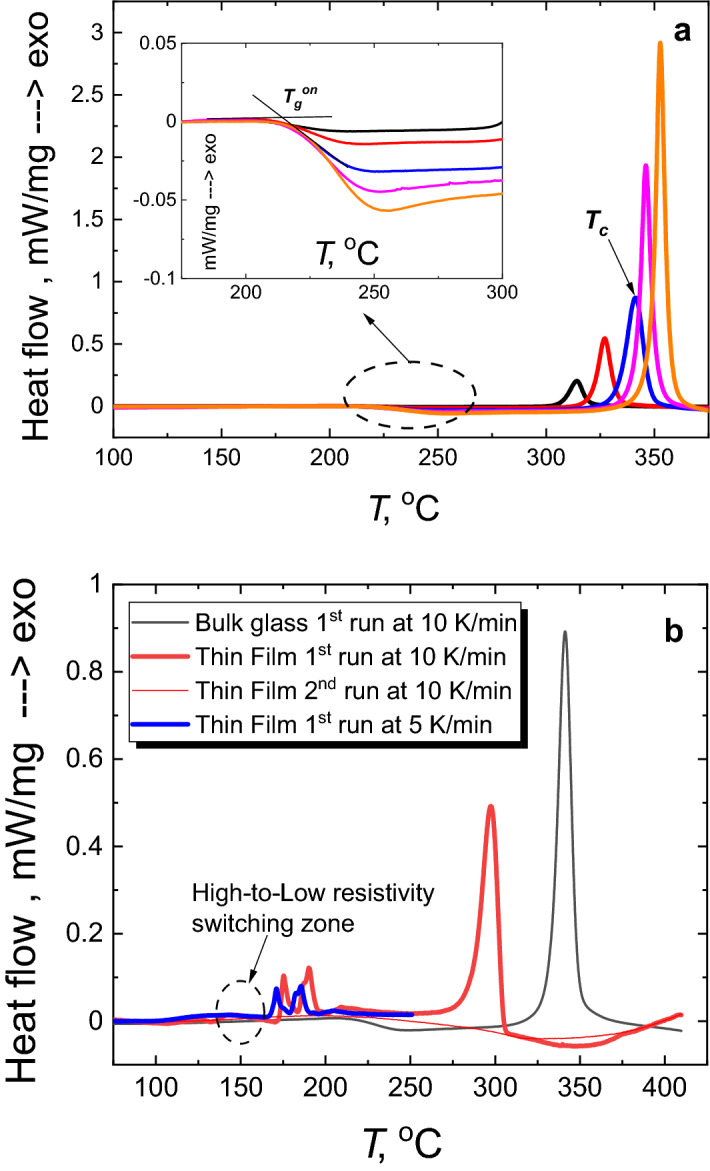
DSC curves of as-prepared bulk Ge_15_Sb_40_S_15_Se_15_Te_15_ glass and thin film. (**a**) DSC signals for the bulk samples were recorded with 2 (black), 5 (red), 10 (blue), 15 (magenta) and 20 (orange) K/min heating rates. They show glass transition range (insert) and exothermal crystallization peaks above 300 °C. (**b**) DSC curves of thin Ge_15_Sb_40_S_15_Se_15_Te_15_ films scraped out of glass substrate show low-temperature shift of main crystallization peak and additional crystallization peaks within ~ 160–200 °C range.

**Figure 4:**
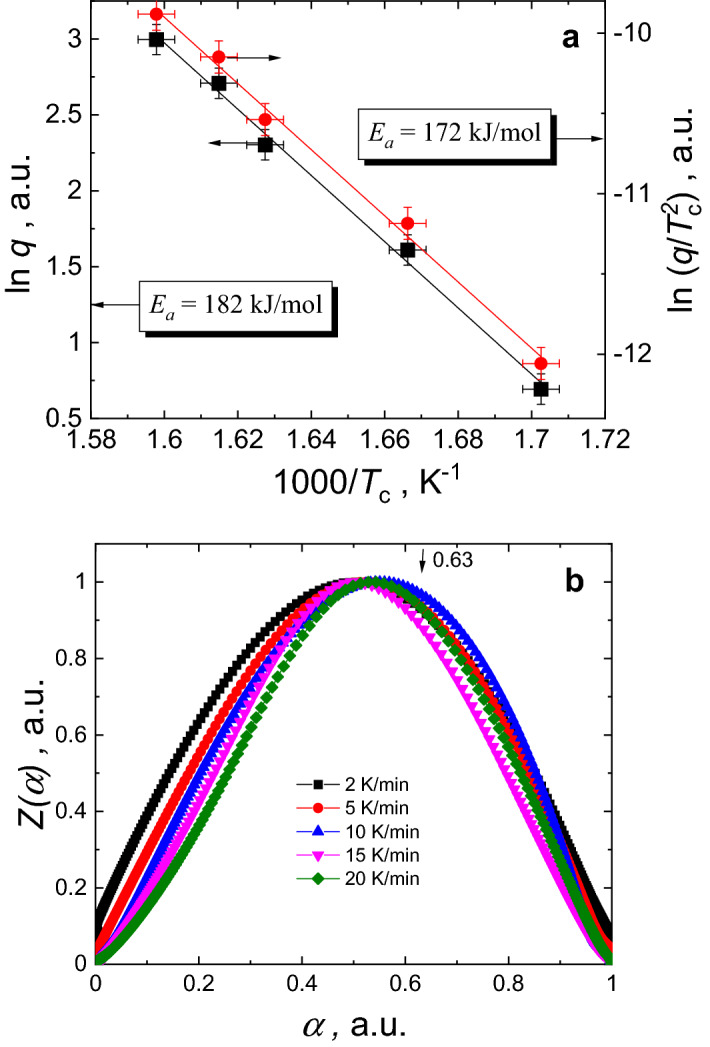
Thermodynamic parameters calculated from DSC data. (**a**) Ozawa’s and Kissinger’s plots for activation energy calculations. (**b**) Probe *z*(*α*) curves, calculated from DSC data of the as-prepared bulk Ge_15_Sb_40_S_15_Se_15_Te_15_ glass, showing peak values shifted from 0.63 position required for JMA model to be valid.

The vitreous nature of the prepared bulk Ge_15_Sb_40_S_15_Se_15_Te_15_ glass can be inferred from Fig. 1, showing typical XRD pattern of glassy substance. The IR image of the obtained bulk rod shows a uniformity of the glass throughout the entire volume (insert to Fig. 1). The optical transmittance window of this material extends from ~ 2.5 to ~ 11 μm without any signature of significant impurities (Fig. 2), typical for purified chalcogenide glasses^17,21,43^. The optical gap (*E*_*g*_) of the obtained bulk glass, estimated using fundamental optical absorption edge data (insert to Fig. 2) and Tauc plots in PARAV program^44^, is 0.71 ± 0.01 eV for indirect and 0.76 ± 0.01 eV for direct transitions. According to these values the Ge_15_Sb_40_S_15_Se_15_Te_15_ glass can be classified as narrow-bandgap semiconductor similar to^45^. It is also on the lower side of *E*_*g*_ values reported for Ge–Te or Ge–Sb–Te glass systems^46^. Advantage of equichalcogenide glass is the possibility to tune the optical gap in wider than pure GST ranges by changing S and Se concentration^39^. Moreover, together with quite high thermal stability of ~ 126 K (so-called Dietzel criterion)^47^, determined from 10 K/min DSC heating curve (Fig. 3a) as the difference between the crystallization peak temperature (*T*_*c*_ = 341.3 °C) and the onset of glass transition temperature (*T*_*g*_^*on*^ = 214.9 °C), this bulk material looks quite attractive for molding and fiber-drawing applications. The obtained value of Dietzel criterion is ~ 70 K on average higher than for binary Ge–Te^48^ and ~ 100 K higher than for ternary Ge–Sb–Te^46^ glass systems. This enables certain applications (in meta-optics or waveguides) of the proposed material, which are not possible or hindered for the conventional GST-based PCMs due to a high crystallization affinity right above *T*_*g*_. The activation energy of crystallization (*E*_*a*_) in the conventional DSC domain can be calculated using Ozawa method^49^ or Kissinger equation^50^ (Fig. 4a). Respective *E*_*a*_ = 182 ± 1 kJ/mol (1.9 eV) and *E*_*a*_ = 172 ± 1 kJ/mol (1.8 eV) values are ~ 0.5 eV smaller than, for example, in pure GST-225 material (which can be barely obtained in a bulk form, though)^51^.

The fraction of crystallized volume *α* during non-isothermal crystal growth from preexisting nuclei can be determined using^52–54^:

1 $$\frac{d\alpha }{dt}=Af(\alpha ){e}^{\left(-\frac{{E}_{a}}{RT}\right)}$$

2 $$\alpha =\frac{1}{\Delta {H}_{c}q}{\int }_{{T}_{0}}^{T}\phi dT,$$

where *ϕ* is the specific heat flow measured with DSC (W/g), Δ*H*_*c*_ is the total enthalpy change associated with the crystallization process, and *q* is the heating rate; the pre-exponential factor *A* and activation energy *E*_*a*_ are kinetic parameters that should not depend on the temperature *T* and *α*. The *f*(*α*) function usually depends on the model used to describe crystallization, of which the most popular is Johnson–Mehl–Avrami (JMA) nucleation-growth model^52–55^. This model, however, is not universal and requires an applicability test for each particular material^52,54^. The easiest way to perform such test in the non-isothermal crystallization conditions was proposed by Malek^52^, who has shown that JMA equation is valid when maximum of probe function

3 $$z(\alpha )=\phi {T}^{2}$$

occurs at around *α* = 0.63 ± 0.02 value. As it is obvious from Fig. 4b, the maximum of *z*(*α*) function calculated from the obtained DSC data is shifted towards lower values, and, therefore, widely used JMA model cannot be directly applied to describe crystallization processes in the investigated bulk material.

Crystallization of thin film scraped with a hard tool from a glass substrate shows a number of disticnct features compare to the bulk glass (Fig. 3b). First of all, the main crystallization peak at ~ 340 °C is shifted to ~ 300 °C in thin film, which is ~ 40 °C lower than in the isocompositional bulk sample. This is due to the influence of extended surface area of the scraped fine powder compare to the bulk sample, which provides abundant seeds for the surface-induced crystallization known to occur at lower temperatures^55^. The second distinct feature is the appearance of additional crystallization peaks at ~ 170–200 °C in powdered thin film samples (Fig. 3b). These peaks are not observed in bulk pieces, probably, because the structure of thin films is more loose and slightly different compare to the bulk glass, since it is assembled from the gaseous phase during evaporation process. The influence of the extended surface area of the scraped films could be an additional reason for the nucleation and crystallization within ~ 170–200 °C temperature range as observed with DSC (Fig. 3b). These crystallization peaks, however, are important to understand phase-change behaviour of equichalcogenide thin films giving an idea on what local arrangements are favored in the bulk, even if the full-scale crystallization cannot occur due to different steric constraints. The peaks look very similar to those obtained for the crystallization of other GST films^46,56^. Third, the glass transition is not visible, although we can speculate from the DSC curves behaviour in Fig. 3b that it is somewhere within 140–160 °C range. This is roughly the temperature range where the resistivity of the investigated thin film first drops by ~ 2 orders in magnitude on heating.

**Figure 5:**
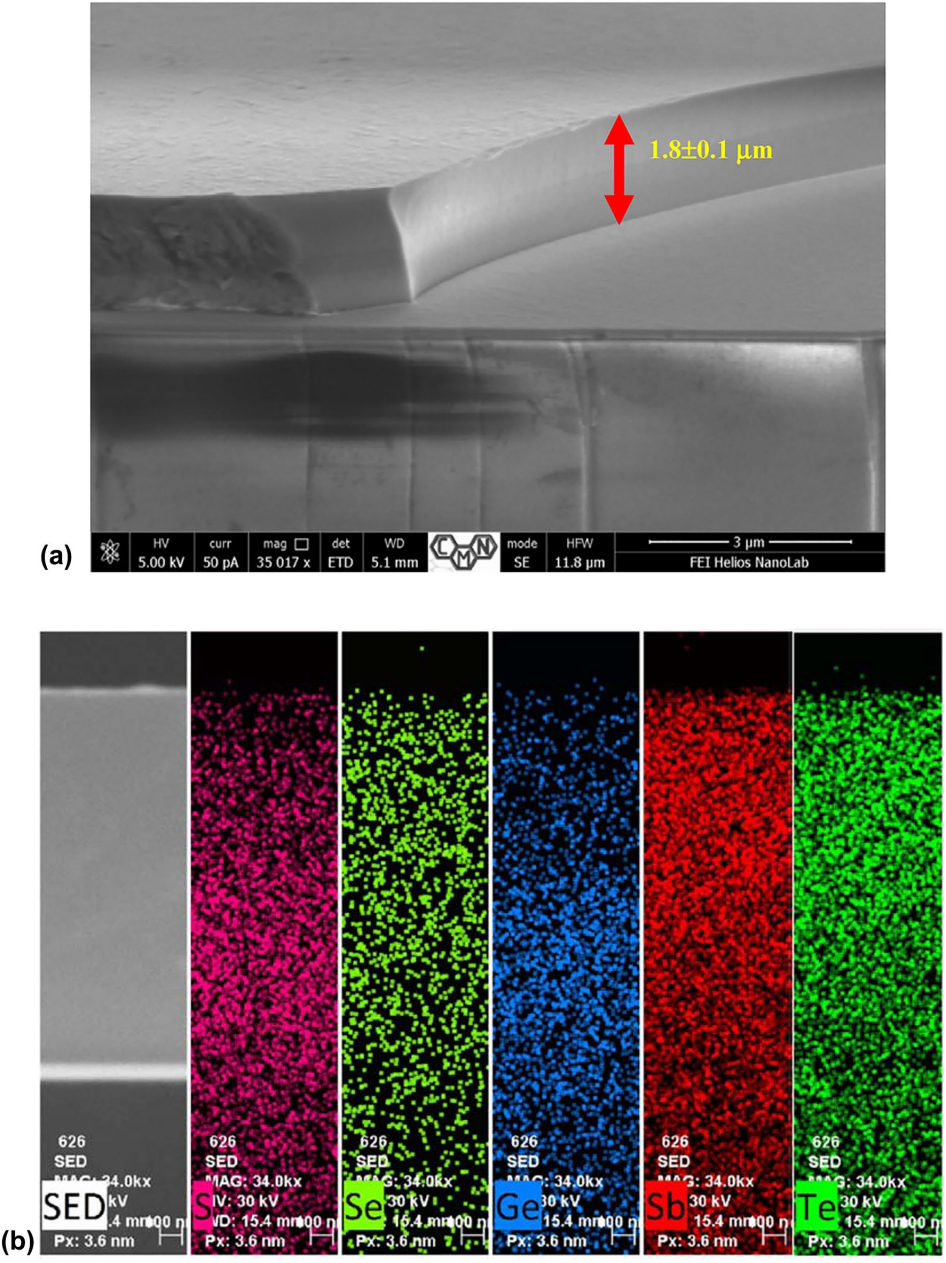
(**a**) The SEM image of the cross-section of amorphous Ge_15_Sb_40_S_15_Se_15_Te_15_ thin film as-deposited on microscopy slide wafer, showing uniform thickness and absence of large-scale inhomogeneities and pores. (**b**) SEM elemental imaging across the cross-section of the film (on the very left), testifying more or less uniform distribution of chemical elements throughout the entire film thickness.

**Figure 6:**
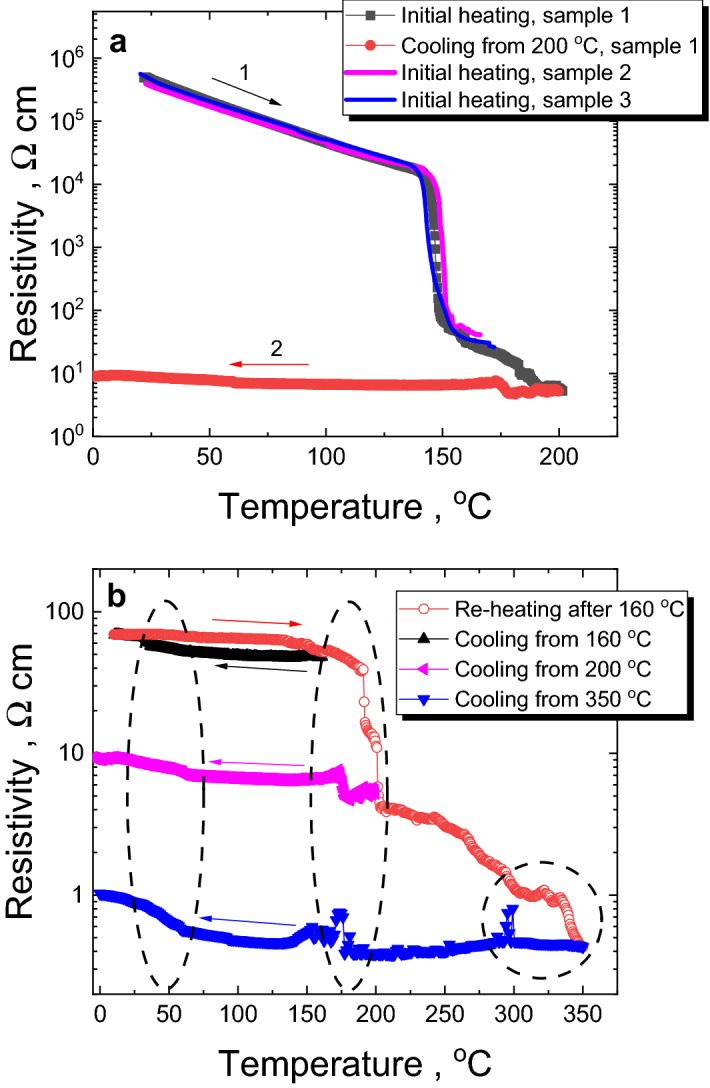
(**a**) Temperature dependence of resistivity measured in heating (step 1) and cooling (step 2) modes with 5 K/min rate for a fresh Ge_15_Sb_40_S_15_Se_15_Te_15_ thin film, deposited on high-density Al_2_O_3_ ceramic substrate with interdigitated electrodes (sample 1), shows rapid switching between High- and Low-resistivity states at ~ 145 °C. To demostrate the influence of small (within 3 at.%) variation in composition on the phase change effect, the temperature dependences of resistivity in heating mode (5 K/min) are shown for the samples 2 and 3 obtained in different synthesis using similar technique and parameters. (**b**) If the previously heated to 160 °C sample is cooled (black) and re-heated again (red) up to 350 °C, a number of features can be observed on resistivity *vs* temperature dependences upon heating and cooling (circled regions).

The typical SEM image of ~ 1.8 μm thick film deposited by thermal evaporation in a vacuum is shown in Fig. 5a. The obtained film is quite uniform in thickness without visible pores or phase separated regions. The composition of the film as obtained from EDS analysis shows about ± 3 at% deviation from the bulk nominal, which is remarkably small considering a 5-component material deposited via thermal evaporation route. A slight gradient in the elements’ concentration can be also noticed from the cross-section elemental analysis in Fig. 5b. However, several different syntheses and measurements on multiple samples show that the observed phase-change memory effect is barely sensitive to such compositional imperfections (Fig. 6a). The use of magnetron sputtering or co-evaporation technique can further improve the quality of the film, but the idea here was to prepare PCM using a cheap and simple method, which can be scalable and comparable with other popular inexpensive technological processes used to obtain chalcogenide materials for various applications. Annealing of the film at 160 °C did not alter much the chemical elements distribution or composition, and no evidences of structural erosion or ~ μm size crystallites formation at this temperature could be obtained through SEM and EDS analysis.

**Figure 7:**
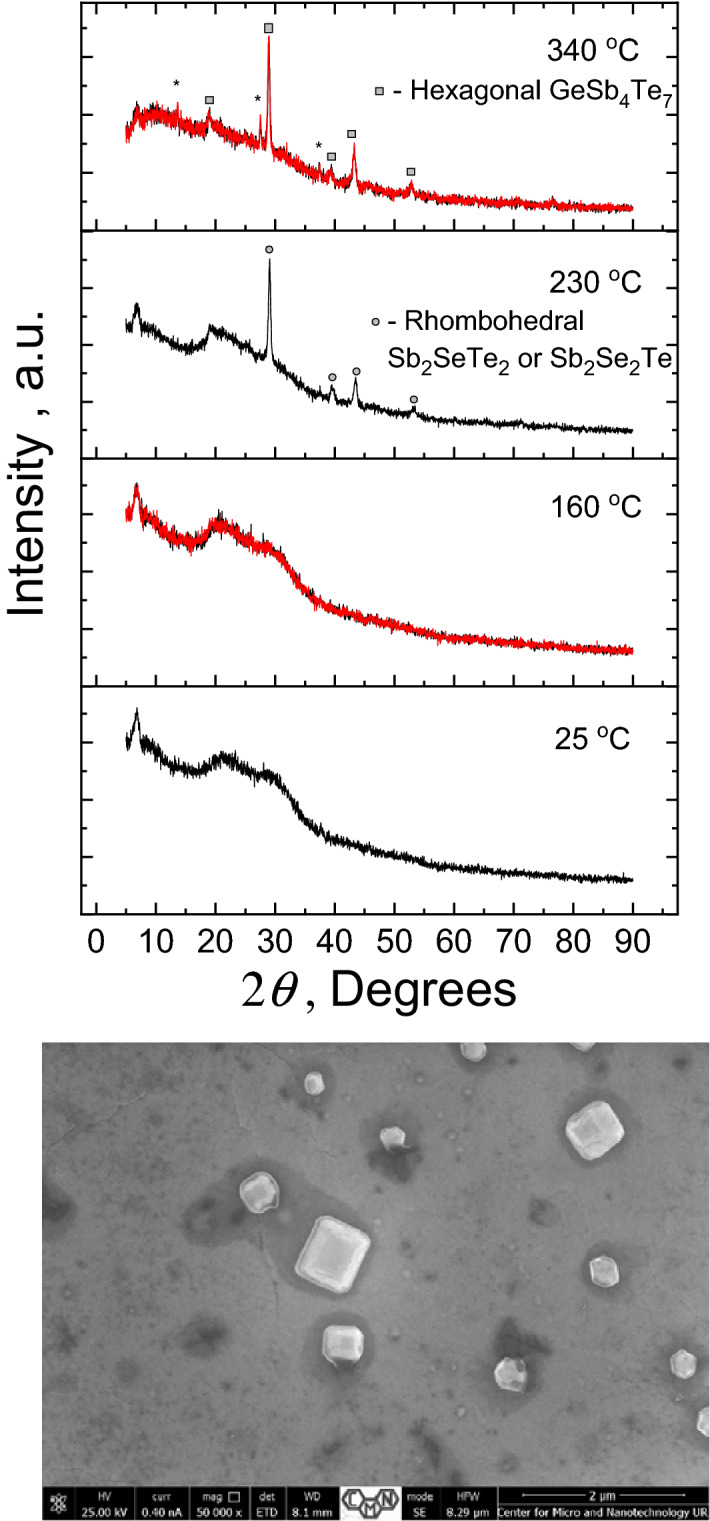
In situ XRD studies at different temperatures. The patterns of Ge_15_Sb_40_S_15_Se_15_Te_15_ thin film deposited on microscopy slide, recorded at different target temperatures (each achieved with heating ramp of 5 K/min), are featureless until ~ 175 °C. Bottom panel shows SEM image of crystallites formed on the surface of the film after annealing at 340 °C.

Resistivity measurements of Ge_15_Sb_40_S_15_Se_15_Te_15_ film deposited on high-density Al_2_O_3_ ceramic substrate with interdigitated metallic electrodes are presented in Fig. 6. The resistivity of the film heated at a constant rate of 5 K/min shows exponential temperature behaviour typical of semiconducting material until ~ 140 °C (Fig. 6a). After this threshold temperature, which though is much lower than *T*_*g*_ ~ 215 °C of bulk samples as obtained from DSC (Fig. 3), the resistivity suddenly drops by several orders in magnitude. Similar drops in resistivity was observed for GeTe at ~ 190 °C^57^, GeSb_2_Te_4_ at ~ 145 °C^57^, Ge_2_Sb_2_Te_5_ at ~ 145 °C^4,56^, Ge_2_Sb_2_Se_2_Te_3_ at ~ 170 °C^4^, Ge_2_Sb_2_Se_4_Te_1_ at ~ 200 °C^4^. In-situ XRD patterns recorded for Ge_15_Sb_40_S_15_Se_15_Te_15_ thin film at different temperatures (each reached with a 5 K/min heating rate) show the absence of a significant crystalline phase at 160 °C temperature (Fig. 7) as there are no visible crystalline reflexes in XRD signal even after 1 h at 160 °C (the curves recorded with 1 h interval are overlapped in Fig. 7), the patterns being very similar to the XRD of the initial amorphous film recorded at 25 °C. According to the obtained XRD temperature-dependent data (Fig. 7), noticeable crystalline reflexes in the investigated thin films are observed above ~ 200 °C, which is consistent with the observation of crystallization peaks in DSC scans of thin films (Fig. 3b). So, we can conclude that the observed abrupt changes in resistivity at 140–160 °C are caused by changes in the structural arrangement at nanoscale towards the nuclei/small crystallites formation, which size/ordering is not enough to give a strong reflex in XRD pattern or significant heat flow signal in the DSC experiments. This effect can be also explained by “amorphous-amorphous” transition, using Peierls distortion mechanism proposed for PCMs as interplay between the short and long bonds, which ratio determines the electronic gap^58^. Crystalline reflexes appeared in the XRD pattern of thin films heated to 230 °C (Fig. 7) can be attributed to GeSb_4_Te_7_, Sb_2_Te_3_, Sb_2_SeTe_2_ or Sb_2_Se_2_Te crystalline phases according to PDF database^59^ and other studies in GST-like PCMs, showing these phases to originate from rhombohedrally deformed cubic symmetry in the arrangement of chalcogens and Ge/Sb atoms^60–63^. It should be noted that GeSb_2_Te_4_ and Sb_2_SeTe_2_ compounds are reported as perspective topological insulator materials^62,64^, which makes the investigated equichalcogenide film even more appealing. Additional reflexes in the XRD patterns recorded at higher than ~ 300 °C temperatures (like at 340 °C, Fig. 7) can be caused either by the oxidation processes or the formation of hexagonal crystalline phase similar to other PCMs^51,65–67^. Because the oxidation processes at below 350 °C temperatures are considered to be a minor effect in this type of materials^66^, affecting only very top surface layers of the film according to our previous studies in GST^68^, the hexagonal phase formation looks more plausible source of additional XRD peaks. Possible hexagonal phases can be identified as GeSb_4_Te_7_, GeSTe or similar crystals using PDF database^59^. The crystallites can be seen on the SEM image of thin-film surface after the annealing at 340 °C (Fig. 7).

The resistivity behaviour above 160 °C is shown in details on Fig. 6b during re-heating of the sample initially cooled from 160 °C. Two drops in resistivity are visible on the heating curve at ~ 200 °C and above ~ 300 °C. These peculiarities can be directly correlated with the above XRD data, so that the formation of extended cubic-type crystallites can be considered as a reason for the resistivity drop at ~ 200 °C, while the transition to a stable hexagonal structure would be responsible for the resistivity change in ~ 300–340 °C temperature range. It agrees with DSC data recorded for bulk and thin film samples, showing crystallization peaks within these temperature ranges (Fig. 3). The proposed structural changes in the investigated equichalcogenide PCM during heating are consistent with the known mechanisms of phase changes in other GST materials, which are based on Peierls distortions^58^ and a sequence of amorphous-to-metastable cubic followed by metastable cubic-to-stable hexagonal phase transitions^69–72^.

On cooling from 350 °C, three different regions at 300 °C, 170 °C and 50 °C with peculiarities in resistivity temperature dependence can be noticed (circled region in Fig. 6b). They are not accompanied by any changes in the heat flow as the DSC cooling curves of thin film samples were smooth (not shown). Possible explanation can be associated with the residual processes related to the above-mentioned phase transitions observed in GST-based PCMs, as well as with specific features of chalcogen counterparts, such as glass transition temperatures of pure Se and Te or various phase transformations in pure S and Se^73,74^. It is interesting to note, that on cooling from 350 °C the investigated crystallized equichalcogenide material exhibits a negative temperature coefficient of resistance within 300–200 °C range of temperatures, which is proper to (semi)metals. Then it changes back below 200 °C to the positive temperature coefficient character to semiconductors.

**Figure 8:**
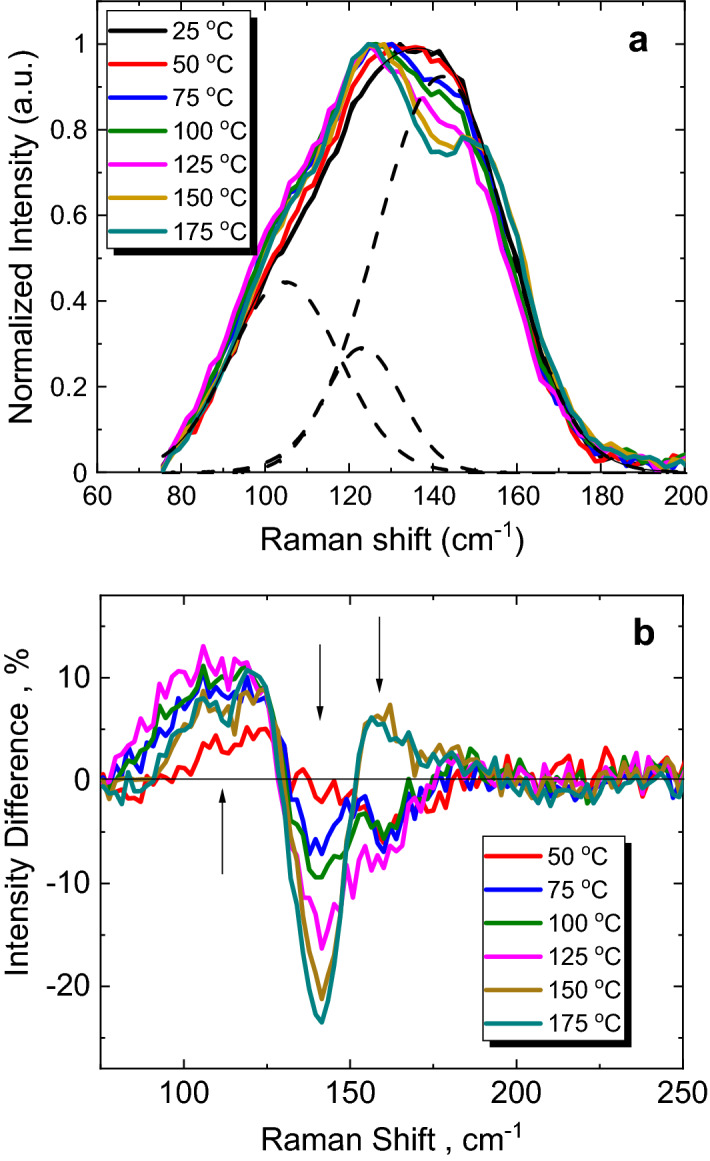
(**a**) Unpolarized Raman spectra of amorphous Ge_15_Sb_40_S_15_Se_15_Te_15_ thin film recorded at different temperatures show typical pattern of Te-based complexes (they are dominated in Raman signal of the investigated glasses). The example of unrestricted Gaussian fit (dashed lines) performed for the signal at 25 °C shows features common to other GST materials. (**b**) Difference between the Raman spectra at indicated temperatures and the 25 °C temperature one gives important information on the temperature-induced transformations in studied material.

Nevertheless, our primary interest is a switching between High- and Low-resistivity states at below ~ 160 °C, where no significant crystalline reflexes are yet observed in the XRD patterns of the films. To shed more light on these structural rearrangements during heating, the Raman data recorded in situ for the amorphous thin film at different temperatures are analyzed (Fig. 8). The unrestricted Gaussian fit of the Raman spectra taken at room or close-to-room temperatures reveals the main features at ~ 105 cm^−1^, ~ 124 cm^−1^ and ~ 142 cm^−1^ (Fig. 8a), which are typical for Raman spectra of amorphous GST materials^75–78^. In the case of pure GST PCMs, the features within ~ 80–100 cm^−1^ are normally associated with dominant contribution from Γ_3_(E) mode of rhombohedrally deformed rocksalt structure (usually observed in α-GeTe single crystal)^79^ and bending modes of GeTe_4_ tetrahedra^77^. The ~ 125–135 cm^−1^ band is attributed to A_1_ mode of corner-shared GeTe_4_ tetrahedra and lighter Ge_2_Te_3_ complexes^77,78^. The band at ~ 145–155 cm^−1^ is either associated with a stretching mode of SbTe_3_ pyramids (compare to Raman spectra of Sb_2_Te_3_)^77^ or with the defective octahedral coordination of Sb atoms^75^. A contribution of edge-shared GeTe_4_ tetrahedra vibrational modes to Raman spectrum of amorphous GST-225 is expected at ~ 168 cm^−1^^75,77^. This mode may also convolute with the A_1g_(2) mode of hexagonal Sb_2_Te_3_ (~ 165 cm^−1^)^75,77^ and Sb–Sb bonds vibrations if present^75,77,80^. Obviously, in the case of equichalcogenide PCMs the contribution from complexes where one or more Te atoms are substituted with Se and/or S are expected in all of these regions. Therefore, the exact assignment of Raman bands in this material demands extensive theoretical calculations and would be also complicated by the overlap with various bending vibrations caused by the mixed S/Se/Te chalcogen-containing complexes in the structure. Moreover, the Raman activity of Te-based complexes is generally much higher than Raman activity of sulfides and selenides, which might be a reason we cannot observe with confidence the pure GeSe(S)_4/2_ tetrahedral or SbSe(S)_3/2_ pyramidal units (if exist) having their signatures in 175–500 cm^−1^ range of spectrum^81,82^ at the low laser intensity used to collect present Raman data. Also, it explains partial similarity of the obtained Raman signal with Raman data obtained earlier for pure GST materials^75–78^. On the other side, the increased 785 nm laser intensity in Raman would lead to strong photo-induced changes in the investigated films, which would distort their structure and make it impossible to catch the temperature-induced changes. Nevertheless, some qualitative conclusions can still be drawn from the differences between the Raman signal recorded at higher temperatures and room temperature spectrum (Fig. 8b). It can be noticed (under a reservation that Raman spectra were normalized, of course), that the relative intensity of the band at ~ 90–125 cm^−1^ gradually increases, while intensities of the bands at ~ 140 cm^−1^ and ~ 160 cm^−1^ decrease up to the threshold temperature ~ 140 °C (Fig. 8b). This recorded temperature behavior of Raman signal in the investigated equichalcogenide PCMs qualitatively resembles the one observed for other GST PCMs^70^. Further heating of the equichalcogenide film above ~ 140 °C leads to an opposite behavior of the band at ~ 160 cm^−1^ and opposite trend in 90–120 cm^−1^ region (Fig. 8b). So, we argue that structural changes responsible for the High-to-Low resistivity drop at threshold temperature ~ 140 °C are somehow related to structural transformations which have their Raman signatures at 100–120 cm^−1^ and ~ 160 cm^−1^. If one assigned the Raman signal in 90–120 cm^−1^ range with rhombohedrally deformed rocksalt structure and in 160–165 cm^−1^ range with Raman-active A_1g_(2) mode of hexagonal Sb_2_Te_3_ phase and/or Sb-Sb bond vibrations^75,77,80^, the conventional "umbrella-flip" mechanism similar to GST^70^ can be used to explain Raman changes in the investigated PCMs. According to this mechanism, the amorphization-crystallization structural transformations are envisioned as mutual reorganization between the well-defined Ge- and Sb-based rigid building blocks, which arrange themselves into rocksalt-type structure where chalcogen atoms form one face-centred-cubic (f.c.c.) sublattice and the Ge/Sb atoms both form the other f.c.c. sublattice with a significant amount of vacant sites^70–72^. When the as-deposited amorphous GST is subjected to a thermal treatment slightly below the glass-transition temperature, the long-range ordering of these rigid blocks occurs leading to strengthened interblock interactions and flip of Ge atoms into the octahedral positions. This is believed to be a diffusionless process, where the rupture of strong covalent bonds within the rigid blocks is not required, which means that material does not have to be transformed into a truly liquid/supercooled liquid state. The converse process, amorphization, is associated with externally-induced (light, temperature) weakening of interblock interactions, which allows the block structure to relax so that the bonds shrink and Ge umbrella-flips into its preferred tetrahedral coordination^70^. This mechanism is also concominant with Peierls distrotions idea used to explain phase-change memory behaviour in PCMs^58^.

So, we can put forward a hypothesis that process responsible for the High-to-Low resistivity switching in equichalcogenides at 140–150 °C proceeds towards the distortion of corner-shared Ge(Te,Se,S)_4_ tetrahedra and Sb(Te,Se,S)_3_ pyramids (including those modified by Sb/Ge–Ge/Sb bonds) with further their rearrangements into Ge(Te,Se,S)_6_ octahedra (Γ_3_(E) mode of single-crystalline α-GeTe with rhombohedrically deformed rocksalt-type structure is observed at 98 cm^−1^^79^) and hexagonal Sb_2_Te_3_—like environment, possibly involving Sb-Sb homopolar bonds^75,77,80^. Such hexagonal environment for Sb atoms can be illustrated at the example of trigonal (R3m space group) structure of Sb_2_SeTe_2_ identified in the annealed film with XRD. It consists of two inequivalent Sb^3+^ sites: Sb^3+^ bonded to six Te^2−^ atoms to form SbTe_6_ octahedra that share corners with three equivalent SbTe_3_Se_3_ octahedra and edges with nine SbTe_6_ octahedra; and Sb^3+^ bonded to three equivalent Te^2−^ and three equivalent Se^2−^ atoms to form distorted SbTe_3_Se_3_ octahedra that share corners with three equivalent SbTe_6_ octahedra, corners with three equivalent TeSb_3_Se_3_ octahedra, edges with three equivalent TeSb_3_Se_3_ octahedra, and edges with nine SbTe_6_ octahedra^83^.

**Figure 9:**
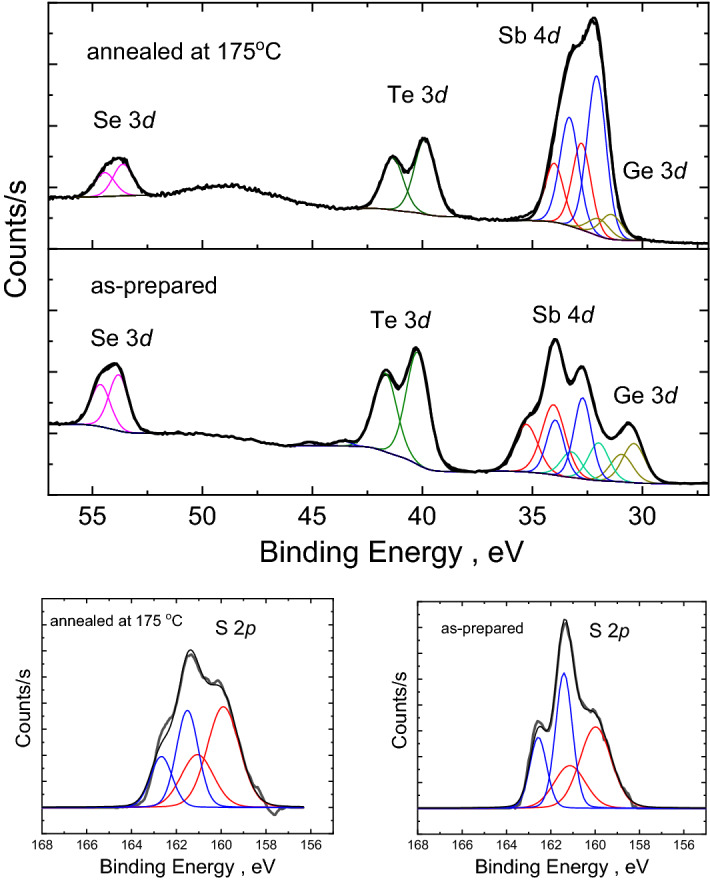
XPS results (bold line—experimental curve; thin lines—fitting components). Comparison of the XPS spectra, recorded for Ge_15_Sb_40_S_15_Se_15_Te_15_ thin film in as-prepared amorphous and annealed at 175 °C states, shows significant difference for Sb, Ge and S core levels. This allows to further refine the mechanism proposed on the basis of Raman and XRD studies.

Along with “umbrella-flip” and Peierls distortion mechanisms occurring at lower temperatures, some transformations with S and Se constituents are also possible in equichalcogenide films upon further heating. Raman spectra cannot provide such an information, since they are dominated by Te-based complexes not showing the S- or Se-based units due to the low intensity of the probe laser. Therefore, the behaviour of sulfide and selenide complexes was assessed through XPS analysis of the as-prepared and annealed at 175 °C films (Fig. 9, Table 1). From the obtained fitting of the overlapped XPS signals (Table 1), it can be concluded that Ge 3*d* core level of the annealed films shifts significantly (~ 1 eV) towards higher BE values, while Sb 4*d* and S 2*p* core level components undergo considerable intensity redistribution. It is known, that chemical shifts in the XPS peaks depend on the electron density distribution around probed element, which is determined mainly by electronegativity of neighbors, their electronic configuration and charge state/coordination. Within such approach, each separate doublet appearing in the fit of the experimental XPS core level spectrum corresponds to a specific chemical environment (structural fragment) of the probed element and its electronic configuration. Some structural fragments, especially in multicomponent compounds, may give close chemical shifts and, thus, cannot be unambiguously resolved by fitting procedure. Then such fragments are fitted with one doublet of increased *fwhm* value as it is done for the investigated equichalcogenide samples (Table 1). Although we cannot identify the exact environment from such fitting as in the case of binary or ternary chalcogenides^84–86^, we believe it is still possible to assess the neighbourhood, coordination and/or charge state of the probed atoms. The high-BE shift of Ge 3*d* core level in the annealed film would correspond well to the Ge switched to octahedral positions from the tetrahedral ones present in the amorphous state. The low-BE shift of the Sb 4*d* core level in the annealed film can be understood if one considers intensity redistribution among two resolved doublets in S 2*p* core level, showing an increase in low-BE S-II component (Fig. 9, Table 1). Then, the mechanism of temperature-induced transformations in the equichalcogenide thin films can be proposed as follows. The initial structure of thin film contains a significant amount of SbS_3_, SbSe_3_ or mixed Sb(Se,S)_3_ pyramids, which contribute to Sb-I and partially Sb-II high-BE doublets (Fig. 9, Table 1)^81,85,86^ due to higher electronegativities of S and Se (2.58 and 2.55, respectively) compare to Te (2.10)^87^. This means that Te prevails in the environment of Ge atoms in the as-prepared films, explaining lower BE for Ge 3*d* core level peak in the as-prepared state (Table 1). Upon annealing, S and Se prefer Ge atoms, substituting Te in the Ge environment, which together with a possibility to flip into octahedral positions as per “umbrella-flip” mechanism mentioned above would explain a significant increase in BE of Ge 3*d* core-level spectrum (S and Se have much higher electronegativities than Te^87^) and a significant decrease of Raman signal at ~ 140 cm^−1^ responsible for corner-shared GeTe_4_ (or Te-dominated) tetrahedra (Fig. 8b). The S in Ge environment is observed at slightly lower BE than S in Sb environment^84^, which would explain the increase of S-II doublet intensity (Fig. 9, Table 1). Then, the released Sb atoms can participate in the formation of Sb–Sb or Sb–Ge bonds and GeSb_4_Te_7_, Sb_2_Te_3_, Sb_2_SeTe_2_ or Sb_2_Se_2_Te phases observed with XRD upon further annealing. It would also explain the increased intensity of Sb-II and Sb-III low-BE doublets in the Sb 3*d* core-level spectra (again, invoking the electronegativity argument).

**Table 1:** Best fit values of characteristic parameters for XPS core level peaks.

Sample	As-prepared			Annealed		
Core level (component)	BE (eV)	*fwhm* (eV)	*A* (%)	BE (eV)	*fwhm* (eV)	*A* (%)
Ge (3*d*_5/2_)	30.39	1.20	100	31.42	1.14	100
Sb-I (4*d*_5/2_)	34.04	1.24	43	–	–	–
Sb-II (4*d*_5/2_)	32.72	0.93	37	32.77	1.00	35
Sb-III (4*d*_5/2_)	31.99	1.10	20	32.08	1.01	65
S-I (2*p*_3/2_)	161.41	0.89	47	161.44	1.18	20
S-II (2*p*_3/2_)	159.98	1.66	53	159.91	1.50	80
Se (3*d*_5/2_)	53.81	1.04	100	53.61	1.06	100
Te (3*d*_5/2_)	40.22	1.22	98	39.89	1.14	100

The binding energy (BE) position, full width at half maximum (*fwhm*) and area (*A*) of the doublets’ main components are shown for Ge, Sb, S, Se and Te core levels, obtained through the fitting of experimental XPS spectra of as-prepared and annealed at 175 °C films.

So, along with the conventional “umbrella-flip” and Peierls distortion mechanisms the studied equichalcogenide films are characterized by some chemical bonds’ redistribution, involving substitution of Te atoms with S and/or Se in the nearest surrounding of Ge and possible clusterization of Sb at higher temperatures.

## Conclusion

Bulk equichalcogenide Ge_15_Sb_40_S_15_Se_15_Te_15_ glass shows thermal stability and optical properties promising for various applications in photonics and meta-optics. The resistivity of equicompositional thin film shows exponential temperature behaviour typical of semiconducting material until ~ 140 °C and drops several orders in magnitude above this threshold temperature, demonstrating phase-change memory effect. The formed Low-resistivity state remains stable upon cooling from 160 °C or higher temperatures. The extended crystallites are not observed with conventional XRD at 160 °C temperature or lower, suggesting local nanoscale mechanisms responsible for the High-to-Low resistivity switching in these materials at ~ 140–150 °C. Temperature-dependent Raman and XPS studies at or below 175 °C suggest distortion of corner-shared Ge(Te,Se,S)_4_ tetrahedra and Sb(Te,Se,S)_3_ pyramids (including those modified by Sb/Ge–Ge/Sb bonds) with further their rearrangements into Ge(Te,Se,S)_6_ octahedra and hexagonal Sb_2_Te_3_—like environment. Substitution of Te in the nearest environment of Ge atoms with S or Se and, possibly, formation of Sb-Sb/Ge bonds are conceivable on the basis of XPS results upon further heating above 150 °C. According to DSC scans, the heating of the as-deposited thin films above 175 °C leads to the crystallization of several phases at ~ 200 °C and ~ 300 °C. The XRD reflexes of these phases coincide with known reflexes of GeSb_4_Te_7_, Sb_2_SeTe_2_ and Sb_2_Se_2_Te crystallites, as well as some unidentified reflexes presumably corresponding to hexagonal crystalline phases like GeSb_4_Te_7_, GeSTe or similar. The observed phase-change memory effect can be explored in the all-chalcogenide photonic/electronic integrated platforms based on the same equichalcogenide family materials.

## Data Availability

All data are available in the main text or upon a reasonable request from Corresponding Author.
